# Pitfall in necrotizing pancreatitis: Organized hematoma with arteriovenous fistula mimicking a splenic artery pseudoaneurysm

**DOI:** 10.1016/j.radcr.2026.08.061

**Published:** 2026-09-12

**Authors:** Sandra López Gordo, Neus Torra, Ana Bernabeu, Ernest Bombuy, Joan de la Cruz

**Affiliations:** a Department of General and Digestive Surgery, Hospital Universitario de Mataró, Barcelona, Spain; b Human Anatomy and Embryology Unit, Department of Morphological Sciences, Faculty of Medicine, Universitat Autònoma de Barcelona, Barcelona, Spain; c Department of General and Digestive Surgery, Hospital Municipal de Badalona, Barcelona, Spain; d Department of Radiology, Hospital Universitario de Mataró, Barcelona, Spain

**Keywords:** Splenic artery pseudoaneurysm, Necrotizing pancreatitis, Arteriovenous fistula, Interventional radiology, Computed tomography, Pitfall

## Abstract

Splenic artery pseudoaneurysm is a rare vascular complication of necrotizing pancreatitis, carrying a significant risk of rupture and massive hemorrhage. Diagnosis and treatment are essential; however, imaging findings may occasionally mimic other vascular lesions, creating diagnostic challenges. We report the case of a patient with severe necrotizing pancreatitis in whom serial contrast computed tomography (CT) examinations demonstrated a hyperdense splenic hilar lesion in the arterial section, increasing from 22 to 54 mm over a short interval, highly suggestive of a splenic artery pseudoaneurysm. The lesion showed imaging characteristics and anatomical location typically associated with this vascular complication. In the follow-up CT scan, the lesion also filled in the venous phase, creating doubt about the previous diagnosis. Selective angiography further supported the diagnosis by demonstrating nodular opacification at the splenic hilum, and prophylactic proximal coil embolization of the splenic artery was performed. Despite treatment, follow-up multiphasic CT revealed persistent arterial flow beyond the embolization coils and arterial link, this finding led to the indication for splenectomy. Splenectomy ultimately revealed an organized hematoma associated with an arteriovenous fistula rather than a true pseudoaneurysm. Retrospective review of the imaging studies identified atypical features, including predominant portal venous phase and the absence of a clearly defined arterial communication on angiography, findings that may help differentiate these entities. This case highlights an important imaging pitfall in one of the most dangerous vascular complications of necrotizing pancreatitis and underscores the importance of correlating CT, angiographic, and surgical findings when the diagnosis remains uncertain.

## Graphical abstract

**Figure:**
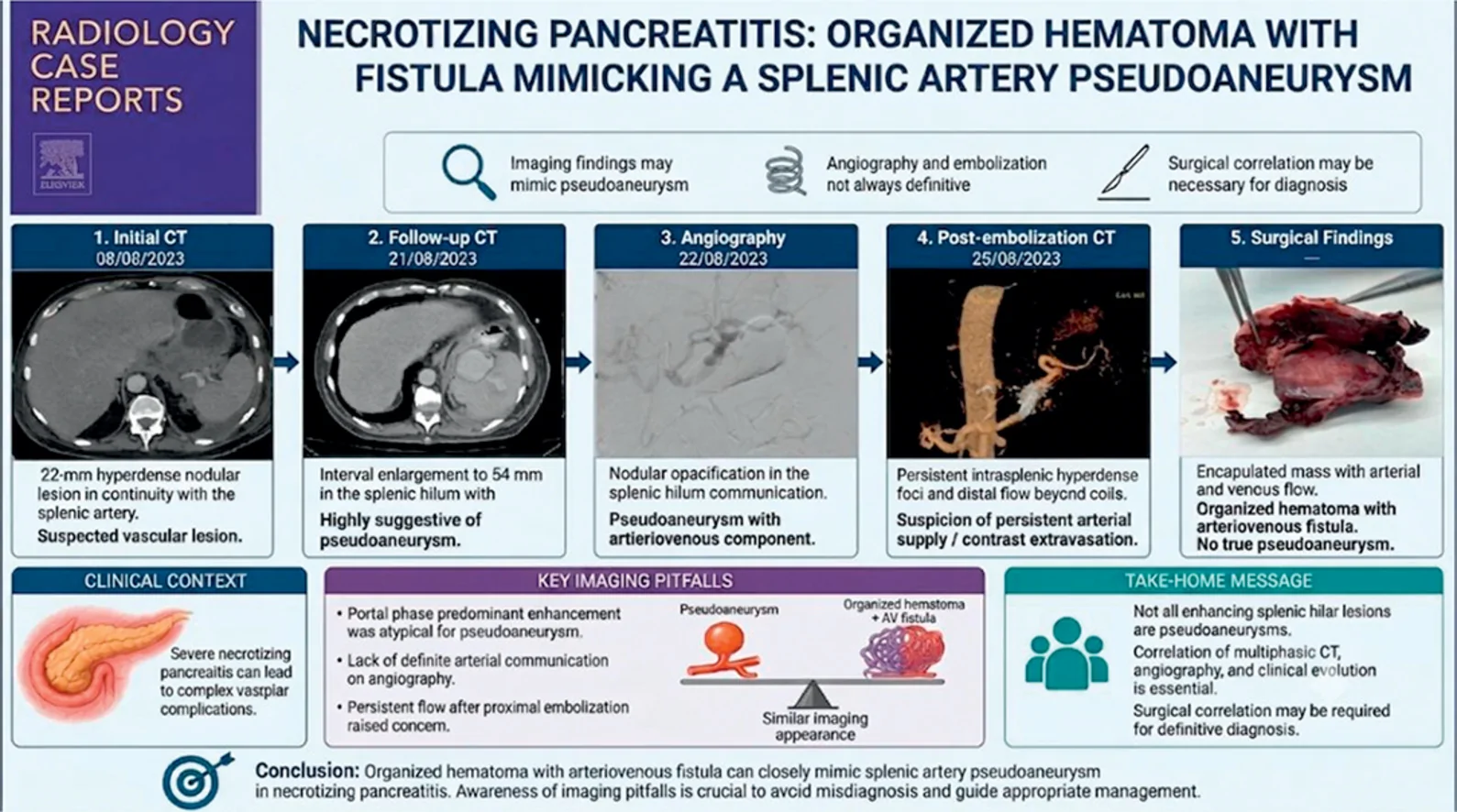

## Case report

A 54-year-old patient was diagnosed and admitted for severe and necrotizing acute biliary pancreatitis. The patient maintained hemoglobin levels of approximately 9 g/dL (range, 8.7-9.4 g/dL). Despite the anemia, the patient remained hemodynamically stable and afebrile throughout the admission, with no evidence of overt gastrointestinal bleeding.

**Fig. 1:**
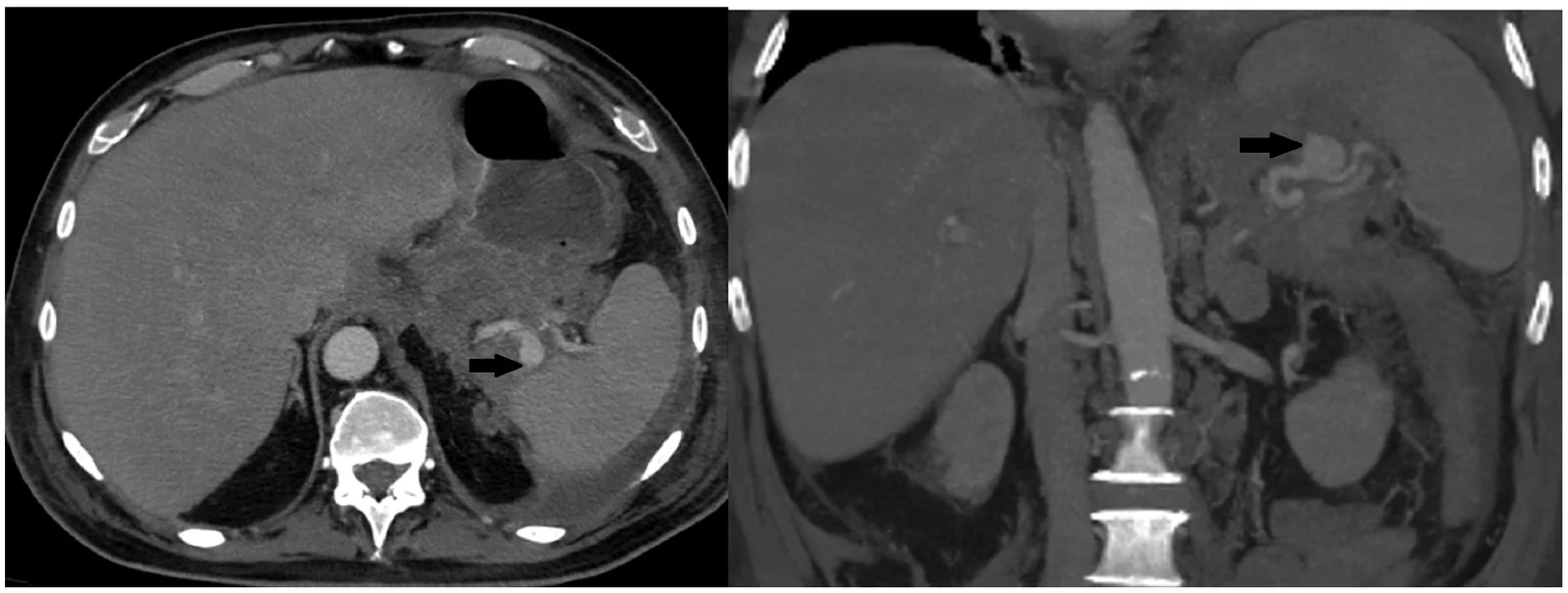
Arterial fase. CT with intravenous contrast performed on August 8, 2023 showing a 22-mm hyperenhancing nodular lesion apparently in continuity with the splenic artery (arrow). The location and arterial enhancement initially raised suspicion of a splenic artery pseudoaneurysm.

During admission, a computed tomography (CT) scan was performed on August 8, 2023, showing a 22 mm hyper dense nodular lesion in the continuity of the splenic artery. The imaging finding mimicked a possible vascular lesion without active bleeding (Fig. 1). A Follow-up CT scan was ordered due to uncertain diagnostic. The CT scans were performed using a multiphase protocol that included baseline, arterial, and portal phases. Acquisition parameters were automatically calculated based on patient characteristics—including height and weight—in accordance with the institution's radiology protocol. No routine delayed phase was included in the protocol.

**Fig. 2:**
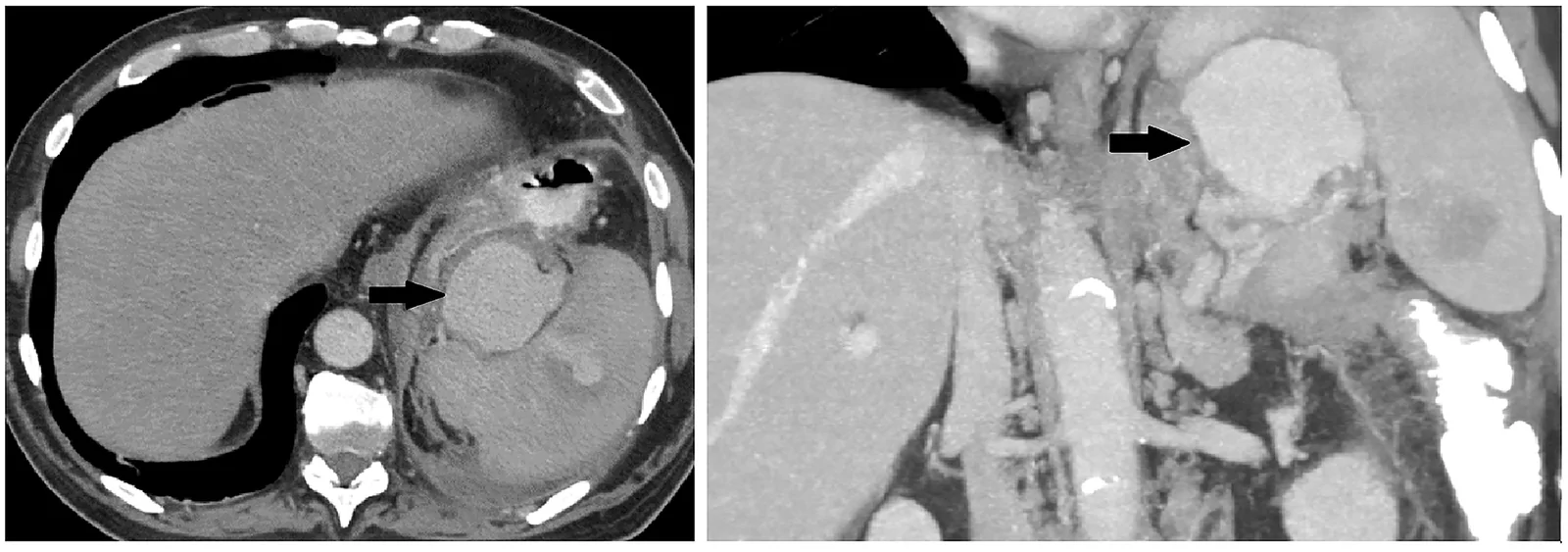
Portal venous-phase contrast-enhanced CT performed on August 21, 2023, showing marked enlargement of the splenic hilar lesion to 54 mm (arrows), with prominent enhancement during the portal venous phase. The persistence of contrast enhancement beyond the arterial phase represented an atypical feature for a classic splenic artery pseudoaneurysm.

The follow-up CT scan on August 21, ordered due to the patient's progressive anemia, showed an increase in the lesion to approximately 54 mm with filling in both the arterial and venous phases. Given the clinical context, this image was indeed suggestive of a possible pseudoaneurysm of the splenic artery (Fig. 2). During the arterial phase, no early opacification of the splenic vein was observed.

**Fig. 3:**
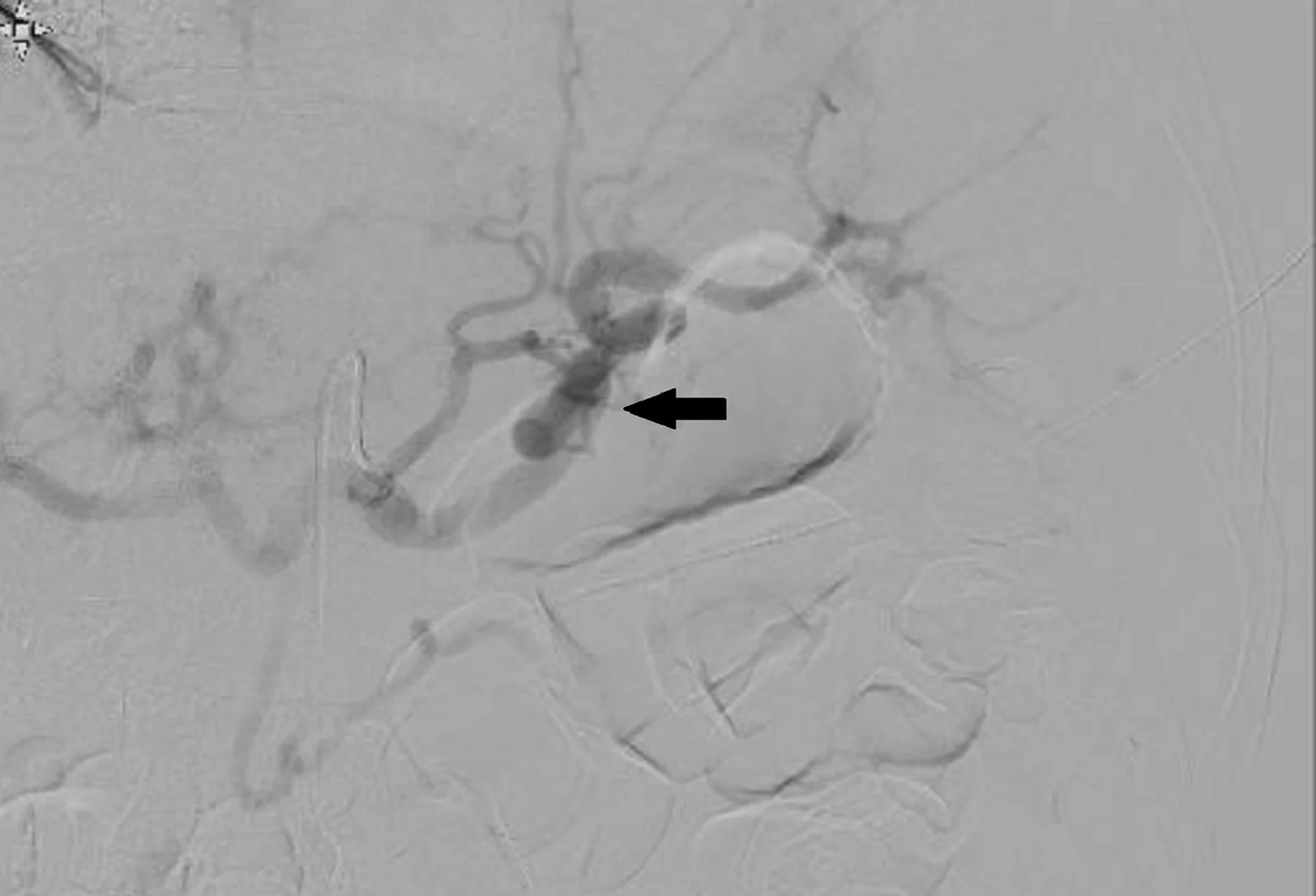
Selective splenic artery angiography demonstrating an image similar to a pseudoaneurysm-like with associated contrast extravasation (arrow). Prophylactic proximal splenic artery embolization with detachable coils was subsequently performed.

Due to the perceived risk of rupture, selective splenic artery angiography was performed on August 22, 2023. Selective splenic artery angiography demonstrated a pseudoaneurysm-like sac at the splenic artery, without a clearly identifiable arterial neck. Contrast extravasation was observed, although no early venous filling or opacification of the splenic vein was identified during the arterial phase. Collateral arterial perfusion of the spleen was also demonstrated. Given the progressive anemia, rapid increase in lesion size, and the high risk of hemorrhage associated with a suspected pancreatitis-related splenic artery pseudoaneurysm, prophylactic proximal embolization of the splenic artery was performed using detachable coils (Fig. 3).

**Fig. 4:**
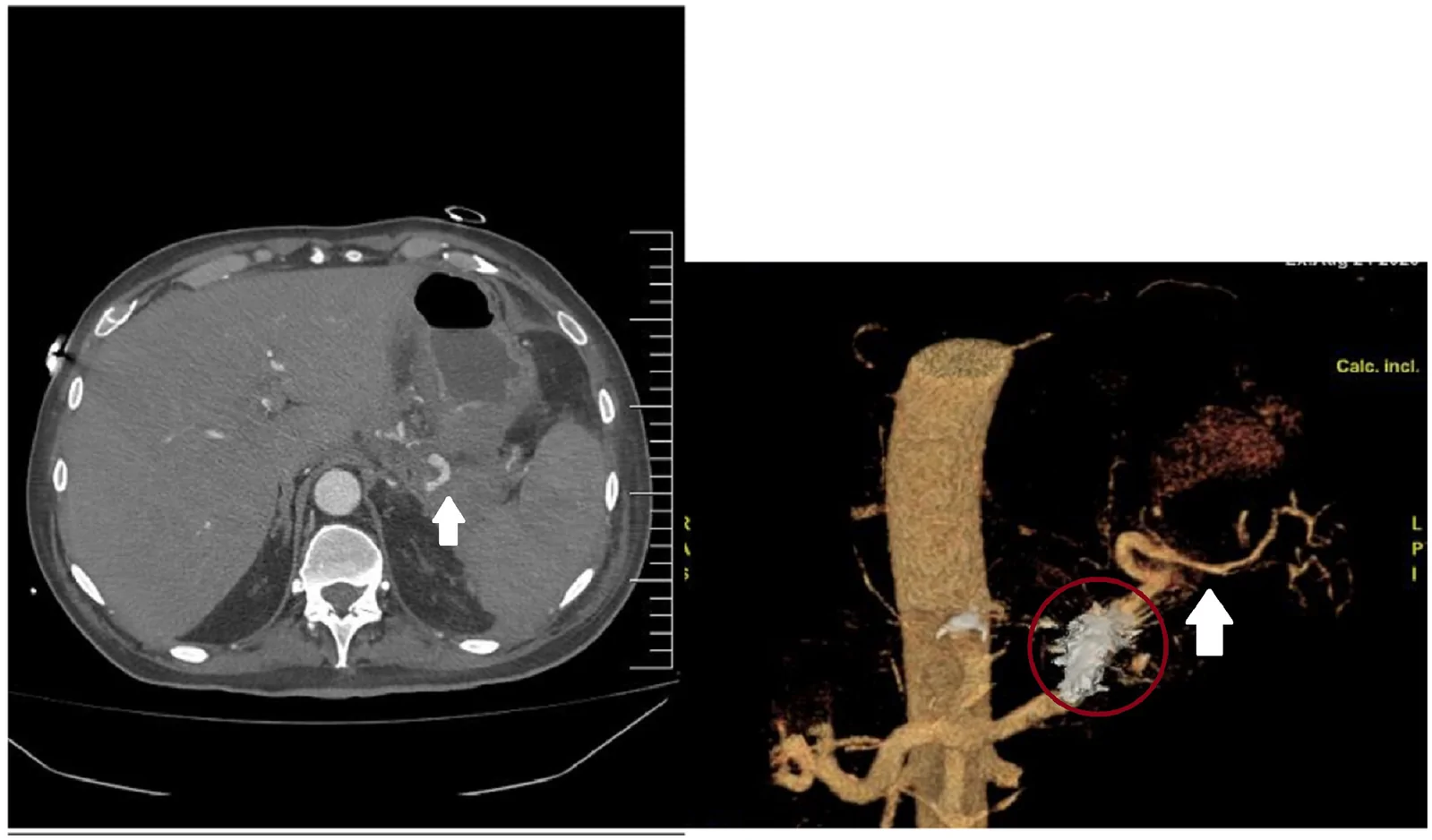
Postembolization multiphasic CT showing persistent hyperdense splenic foci and distal arterial flow beyond the embolization coils with arterial leak. Circle indicating coil embolization of the splenic artery; the arrow points to persistent arterial flow in the splenic artery despite proximal embolization.

Three days later, follow-up multiphasic CT demonstrated persistent hyperdense foci within the spleen and distal arterial perfusion beyond the embolization coils. Although distal splenic perfusion could be explained by the collateral arterial circulation observed angiographically, persistent enhancement of the lesion raised concern that the suspected vascular lesion had not been completely excluded (Fig. 4).

**Fig. 5:**
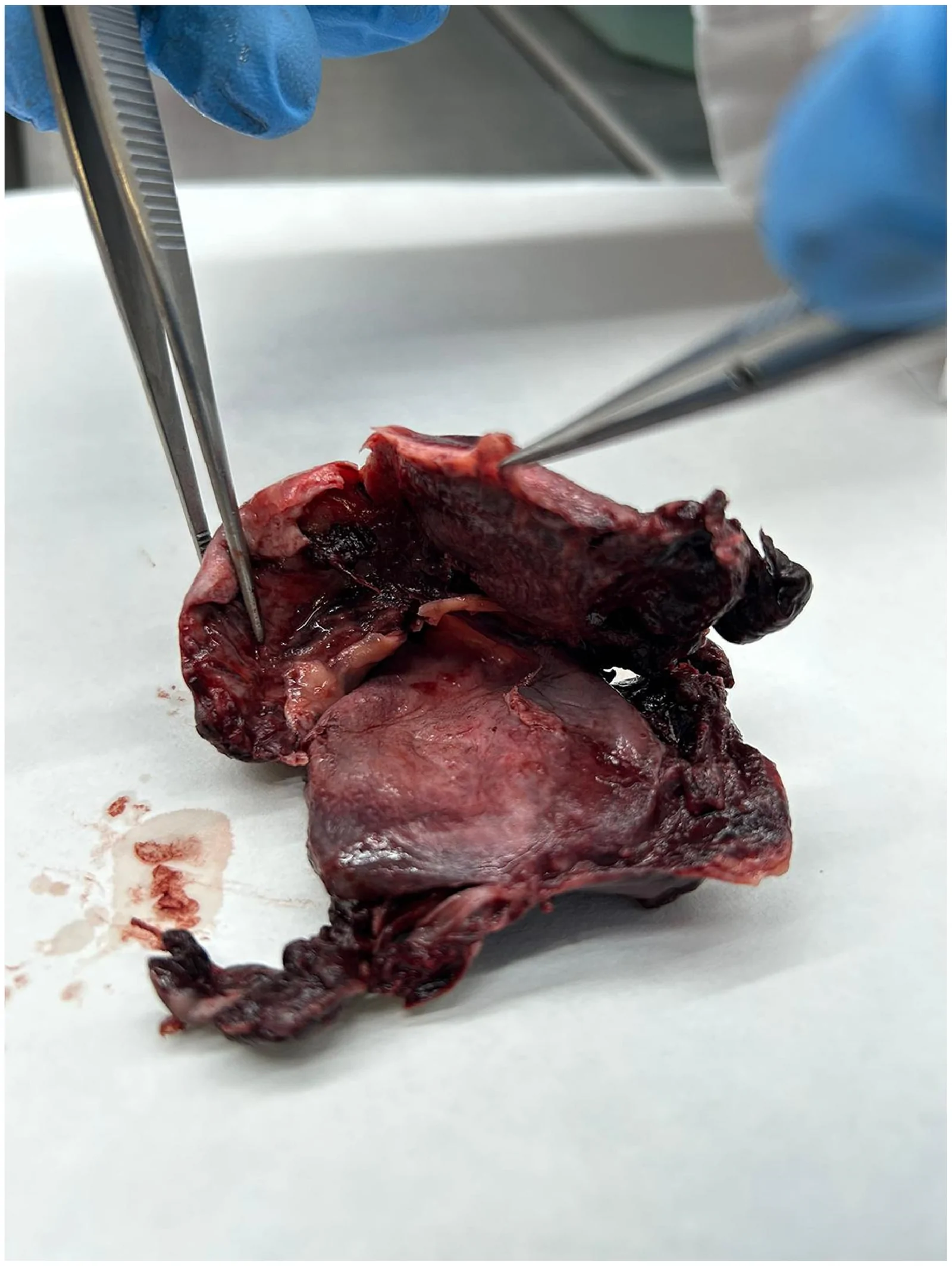
Surgical specimen demonstrating an organized hematoma associated with an arteriovenous fistula. No true splenic artery pseudoaneurysm was identified.

Given the persistent radiological abnormalities and uncertainty regarding lesion exclusion, surgical management was undertaken. Splenectomy was performed. Intraoperatively, surgeons identified an encapsulated mass containing both arterial and venous flow with a well-formed wall. It was oriented as a hematoma formed with arterial and venous flow within it (Fig. 5). The differences between the lesions are detailed in Table 1. Although the exact arterial-to-venous connection could not be anatomically demonstrated, the arterial component appeared to arise from the splenic arterial circulation, whereas venous-phase filling appeared to originate from the splenic hilar vascular territory. Therefore, a direct fistulous communication between the main splenic artery and splenic vein could not be confirmed. On imaging, this complex vascular pattern had mimicked a splenic artery pseudoaneurysm with both arterial and venous filling. This was confirmed in the definitive histopathological analysis in which no true splenic artery pseudoaneurysm was identified (Fig. 5). The definitive histopathological study revealed organized fibrin-leukocyte material forming a mature fibrin thrombus, with no evidence of epithelial or endothelial cellularity.

**Table 1:** Imaging characteristics of vascular and hemorrhagic splenic lesions in pancreatitis.

Entity	CT findings	Enhancement pattern	Angiographic findings	Key distinguishing features
Splenic artery pseudoaneurysm	Well-defined round or oval lesion adjacent to the splenic artery	Arterial-phase enhancement similar to the parent artery; may remain opacified on later phases	Contrast-filled sac communicating with the splenic artery; an arterial neck may be identified	Saccular outpouching lacking the 3 normal arterial wall layers, typically with a neck; often associated with trauma, pancreatitis, or infection
True splenic artery aneurysm	Focal dilatation of the splenic artery; mural calcification or thrombus may be present	Enhancement follows the arterial blood pool. No delayed washout	Fusiform (occasionally saccular) dilatation that is continuous with and part of the arterial wall, without a discrete neck	Preserved 3-layered arterial wall and fusiform shape, distinguishing it from the saccular, neck-bearing pseudoaneurysm
Hemorrhagic pseudocyst	Fluid or heterogeneous collection containing hyperdense hemorrhagic material	Usually no internal arterial enhancement unless an associated vascular lesion is present	No intrinsic vascular filling	Hemorrhagic contents without direct vascular communication
Organized hematoma	Heterogeneous or encapsulated mass. No intrinsic vascular filling on angiography; occasionally shows peripheral blush from granulation tissue	Usually absent or limited internal enhancement; vascularized organized components may produce atypical enhancement	Usually no defined arterial sac or neck; findings may be nonspecific when associated with vascular communication	Organized blood products and fibrous tissue; may mimic a vascular lesion when associated with abnormal vascularization
Arteriovenous communication/fistula	Abnormal vascular structures or an enhancing lesion involving adjacent arterial and venous communication	Arterial enhancement with possible early venous enhancement	Early venous filling during arterial injection and demonstration of an arterial-to-venous communication when identifiable	Early venous opacification is characteristic, although the exact communicating vessels may not always be demonstrated

## Introduction

Severe necrotizing pancreatitis is associated with a wide range of local and systemic complications, including vascular involvement. Among these, visceral artery pseudoaneurysms represent one of the most feared complications because of their high risk of rupture and potentially catastrophic hemorrhage [1,2]. The splenic artery is the vessel most frequently involved, accounting for approximately 60%-65% of pancreatitis-related visceral pseudoaneurysms because of its close anatomical relationship with the pancreas [1,2].

CT with intravenous contrast, is generally the first-line imaging modality for detecting vascular complications in patients with pancreatitis. Typical imaging findings of a splenic artery pseudo aneurysm include a well-defined hyper enhancing lesion demonstrating attenuation similar to the parent artery during the arterial phase [3]. Digital subtraction angiography remains the reference standard for diagnosis and allows simultaneous therapeutic intervention when required [1,4,5]. However, diagnostic interpretation may become challenging in the setting of complex inflammatory, hemorrhagic, and vascular changes related to pancreatitis, where atypical lesions can mimic pseudoaneurysms and lead to diagnostic uncertainty [3,6].

We present a case in which a vascular lesion secondary to necrotizing pancreatitis closely simulated a splenic artery pseudoaneurysm on both CT and angiography. The final diagnosis of an organized hematoma with arterial and venous flow, was established only after surgical exploration and histopathological evaluation. The case illustrates an important imaging pitfall and highlights the value of correlating radiological findings with clinical evolution and surgical outcomes.

## Discussion

Visceral artery pseudoaneurysms are uncommon but well-recognized complications of pancreatitis. Their formation is thought to result from enzymatic digestion and weakening of the arterial wall caused by pancreatic enzymes and inflammatory mediators, ultimately leading to arterial wall disruption and pseudoaneurysm formation [1,2]. Because rupture may occur unpredictably and is associated with substantial morbidity and mortality, prompt diagnosis and treatment are essential [7]. CT angiography plays a central role in diagnosis. A typical splenic artery pseudoaneurysm appears as a round or oval lesion showing arterial-phase contrast filling similar to the parent artery, usually with persistent opacification on delayed images [3]. Nevertheless, image interpretation may become challenging in the setting of extensive inflammation, hemorrhage, and complex peripancreatic collections, where vascular and nonvascular lesions may share overlapping imaging characteristics [6,8].

The present case illustrates an important diagnostic pitfall. The lesion demonstrated several features classically associated with a splenic artery pseudoaneurysm, including a hyperdense nodular appearance, apparent continuity with the splenic artery, progressive enlargement, and angiographic opacification. These findings initially supported the diagnosis of a pseudoaneurysm and justified endovascular treatment. Although spontaneous regression of splenic artery pseudoaneurysms has occasionally been reported, current management recommendations generally favor intervention because of the unpredictable risk of rupture [9].

Endovascular embolization is currently considered the preferred first-line treatment for most non-ruptured visceral pseudoaneurysms and has demonstrated high technical success rates while avoiding the morbidity associated with open surgery [5,10,11]. In our patient, however, several imaging findings were atypical. Enhancement was more prominent during the portal venous phase than expected for a classic pseudoaneurysm, and angiography failed to demonstrate a definitive direct arterial communication. In retrospect, these subtle discrepancies suggested a more complex vascular process.

Regarding the organized hematoma: as the definitive histopathological analysis revealed no endothelium, the arteriovenous communication within the organized hematoma was an entity identified via imaging.

The differential diagnosis of a splenic hilar lesion in the setting of pancreatitis should include organized hematoma, hemorrhagic pseudocyst, arteriovenous fistula, and true or false aneurysmal lesions. Similar diagnostic challenges have been reported in vascular lesions mimicking pancreatic tumors or other hypervascular abnormalities, emphasizing the importance of careful interpretation of enhancement patterns and multimodality assessment [6,12]. Arteriovenous fistulas associated with pancreatitis are rare but recognized complications resulting from inflammatory injury involving adjacent arterial and venous structures. Furthermore, pseudoaneurysms associated with arteriovenous fistulization have also been described, producing atypical arterial and venous filling characteristics [4].

Arteriovenous fistulas related to pancreatitis are rare vascular complications. Their pathogenesis is thought to involve inflammatory erosion of adjacent arterial and venous structures, occasionally associated with splenic vein thrombosis and collateral venous circulation. A splenic arteriovenous fistula secondary to acute necrotizing pancreatitis has previously been reported and demonstrated early venous opacification during arterial-phase imaging, findings that may overlap with those of pseudoaneurysms. Likewise, pseudoaneurysms associated with arteriovenous fistulization have been described in patients with pancreatitis, further increasing diagnostic complexity.

In retrospect, the portal venous phase enhancement, the absence of a clearly demonstrable arterial neck, and the persistence of vascular filling after embolization represented atypical findings for a classic splenic artery pseudoaneurysm and may have reflected the underlying arteriovenous communication. This observation constitutes the principal teaching point of the present case. On the arterial-phase CT scan, the lesion appeared to be a possible arterial pseudoaneurysm, yet showed contrast enhancement during the venous phase—a finding that might suggest an arteriovenous fistula. On the arteriogram, the appearance was also consistent with a pseudoaneurysm, but without venous filling.

Ultimately, surgical exploration established the diagnosis of an organized hematoma associated with an arteriovenous flow rather than a true pseudoaneurysm. This case emphasizes that organized hematomas with abnormal vascular communications may partially reproduce the characteristics of pseudoaneurysms and generate false-positive imaging findings. Although digital subtraction angiography is traditionally regarded as the reference standard for vascular lesion assessment, this case suggest that angiographic findings may occasionally be inconclusive or misleading when complex arteriovenous communications and organized hematomas coexist [1,3].

Careful evaluation of enhancement dynamics across multiple imaging phases, correlation with angiographic findings, and multidisciplinary discussion involving diagnostic radiologists, interventional radiologists, and surgeons are essential when imaging features are equivocal.

## Conclusion

Splenic artery pseudoaneurysm is a rare but potentially fatal vascular complication of necrotizing pancreatitis. However, not all splenic hilar lesions represent true pseudoaneurysms. Organized hematomas associated with arteriovenous fistulas may closely mimic pseudoaneurysms on both CT and angiography, leading to diagnostic uncertainty. Recognition of atypical enhancement patterns and correlation between multimodality imaging findings and clinical evolution are crucial for accurate diagnosis and appropriate management.

## Declaration of generative AI and AI-assisted technologies in the manuscript preparation process

During the preparation of this work, the authors used AI to assist with language editing, grammar correction, manuscript organization, and the preparation of an initial graphical abstract draft. All generated content was carefully reviewed, verified, modified, and approved by the authors. The authors take full responsibility for the accuracy, integrity, and scientific content of the published work.

## Patient consent

Written informed consent was obtained from the patient for publication of this case report and the accompanying images. A copy of the written consent is available for review by the Editor-in-Chief of this journal upon request.
